# Precision Surgery for Glioblastomas

**DOI:** 10.3390/jpm15030096

**Published:** 2025-02-28

**Authors:** Stephen J. Price, Jasmine G. Hughes, Swati Jain, Caroline Kelly, Ioana Sederias, Francesca M. Cozzi, Jawad Fares, Yonghao Li, Jasmine C. Kennedy, Roxanne Mayrand, Queenie Hoi Wing Wong, Yizhou Wan, Chao Li

**Affiliations:** 1 Cambridge Brain Tumour Imaging Laboratory, Academic Neurosurgery Division, University of Cambridge, Cambridge Biomedical Campus, Cambridge CB2 0QQ, UK; jgh45@cam.ac.uk (J.G.H.); ids35@cam.ac.uk (I.S.); fmg39@cam.ac.uk (F.M.C.); jf751@cam.ac.uk (J.F.); yl860@cam.ac.uk (Y.L.); jck52@cam.ac.uk (J.C.K.); rm945@cam.ac.uk (R.M.); qhww2@cam.ac.uk (Q.H.W.W.); yw435@cam.ac.uk (Y.W.); cl647@cam.ac.uk (C.L.); 2 Division of Neurosurgery, University Surgical Cluster, National University Health System, 1E Lower Kent Ridge Road, Singapore 119074, Singapore; 3 Department of Neuro-Oncology Outpatient Physiotherapy, Cambridge University Hospitals, Cambridge CB2 0QQ, UK; 4 Department of Neurological Surgery, Feinberg School of Medicine, Northwestern University, Chicago, IL 60208, USA; 5 Department of Neurosurgery, John Radcliffe Hospital, Headley Way, Headington, Oxford OX3 9DU, UK; 6 Department of Biomedical Engineering, School of Science and Engineering, Fulton Building, University of Dundee, Dundee DD1 4HN, UK

**Keywords:** rehabilitation, prehabilitation, surgical resection, glioblastoma, brain mapping

## Abstract

Glioblastomas are the most common primary malignant brain tumor. Most of the recent improvements their treatment are due to improvements in surgery. Although many would consider surgery as the most personalized treatment, the variation in resection between surgeons suggests there remains a need for objective measures to determine the best surgical treatment for individualizing therapy for glioblastoma. We propose applying a personalized medicine approach to improve outcomes for patients. We suggest looking at personalizing preoperative preparation, improving the resection target by understanding what needs removing and what ca not be removed, and better patient selection with personalized rehabilitation plans for all patients.

## 1. Background

Glioblastomas are the most common and most aggressive form of malignant brain cancer. Unfortunately, we rarely cure these tumors, so we aim to maintain quality of life for as long as possible. As so many trials of new treatments for glioblastomas have failed to demonstrate survival advantages, we need to optimize our existing treatments.

Surgery remains a critically important treatment for glioblastomas. The extent of tumor resection (and, more importantly, the residual tumor volume) is the only prognostic factor we can alter. Removing the entire tumor visible on MRI scans improves survival and maintains good quality of life [1]. However, virtually all patients will progress at the edge of the area removed during surgery. This is because these tumors spread into the surrounding normal brain, and this invasion cannot be seen on conventional MRI. For that reason, there is a lot of interest in supramarginal tumor resection to extend resection into these areas of occult tumors. Despite published benefits of supramarginal resection, these techniques have not become routine due to concerns of injury to the normal surrounding brain. As a result, neuro-oncology guidelines promote ‘maximal safe resection’ [2] without defining what that means, how it should be achieved, and whether it is an appropriate approach for all patients.

**Figure 1:**
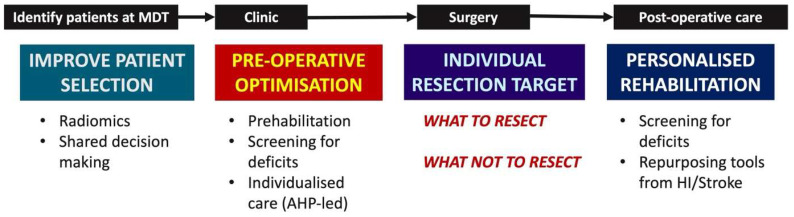
Stages of a patient’s journey suitable for a personalized surgical approach. This is obtained, according to the multidisciplinary tumor board (MDT), through preoperative care, intraoperative management, and post-operative optimization for oncological therapies.

Although many would describe surgery as the most personalized treatment, we believe this is not entirely true. There is significant variability between surgeons regarding resectability, suggesting a need for more objective measures for individualized patients [3]. Personalized medicine means giving the correct treatment to the correct patient at the correct time. It is well appreciated that drug treatments may help some patients but might have detrimental effect on others. The current paradigm of personalized medicine assesses various factors for an individual to determine the optimum patient management. We propose that this approach is needed in surgery. In this paper, we will outline five areas where we can develop personalized surgical care to provide the best treatment for an individual patient. These areas (summarized in Figure 1) are as follows:

1. Preparing patients for surgery.
2. Defining our resection target for individual patients.
3. Understanding what we cannot resect to maintain quality of life.
4. Patient selection: do all patients need maximal, safe resection?
5. Post-operative management of patients to optimize them for adjuvant oncological therapies.

## 2. Preparing Patients for Surgery

Patients with high-grade gliomas tend to be older [4] with the associated issues of frailty and co-morbidities. Glioblastoma patients often have overt motor/speech deficits preoperatively that may not be completely resolved with steroid therapy. As a result, these patients are at a significantly higher risk of developing worse neurological deficits post-operatively [5]. Additionally, there is a very high incidence of neuro-cognitive deficits in this patient group. These have a major impact on quality of life [6]. Unlike low-grade gliomas, patients with glioblastomas do not have the time to recover from post-operative deficits, as decisions on adjuvant oncological therapies depend on good performance status and are made within a short interval of surgery. For that reason, rehabilitation (i.e., treatment after a deficit) may be too late. We know that patients discharged to a rehabilitation facility rather than home have poorer survival [7]. Therefore, we need to optimize our patients preoperatively.

Prehabilitation is a process that helps patients improve their physical and mental health before surgery or cancer treatment. It provides an opportunity for an objective assessment of patient functioning. There is good evidence that prehabilitation before major abdominal surgery improves post-operative outcomes [8]. Unfortunately, evidence for prehabilitation for brain tumor patients is lacking.

There are two areas that need developing for prehabilitation in brain tumor patients:

1. Objective assessment and screening for neurological deficits.
2. Prehabilitation interventions.

### 2.1. Identifying PreOperative Deficits

There are two issues with preparing patients for surgery. The first is identifying deficits; the second is developing interventions to overcome these deficits. Our work mainly focused on identifying these deficits. The DAMSEL project screened patients preoperatively with the EORTC-QLQ30 (version 3) quality of life questionnaire with the brain tumor (BN20) module. We demonstrated this could be done routinely, with data collected on over 1000 patients from two neurosurgical units [9]. It identified a lack of concordance between the clinical problems patients faced (fatigue and cognitive symptoms were the commonest) and what the physician identified (predominantly physical symptoms) [Unpublished Data—Alexis Joannides].

Within the preoperative AHP clinic, assessments were carried out to identify deficits that may affect the individual’s ability to participate in prehabilitation. To reduce the demand on patients, tools incorporating multiple components of ability were used where possible. All measures have been validated for use in similar neurological populations, such as stroke and TBI. In addition to the neuro physiotherapy assessment (power, sensation, proprioception, coordination), the measurement tools routinely included were as follows:

1. 10 m Walk Test (gait, walking speed, dynamic balance).
2. BERG Balance Scale (gold standard balance tool).
3. 9 Hole Peg Test (upper limb function, dexterity, coordination).
4. Montreal Cognitive Assessment.
5. Fatigue Severity Scale.

Any additional assessment tools were chosen through clinical judgment based on the tumor’s location and the patient’s subjective information. The prehabilitation exercise advice depended on what the physiotherapist and occupational therapist deemed safe and feasible based on the outcome of the assessments. Discussion encouraged patients to self-identify ways in which they could participate in exercise before surgery.

### 2.2. Prehabilitation Interventions

The American College of Sports Medicine (ACSM) has produced exercise prescription guidelines, stating that individuals with cancer should aim to complete 150 min of moderate-intensity exercise and at least two sessions of resistance training per week [10]. Gliomas can cause physical neurological deficits, which can affect an individual’s ability to participate in exercise [11]. Although the research volume is limited, early studies have found exercise interventions to be safe, feasible, and effective for people with brain tumors in settings where patients have undergone assessment by a physiotherapist before participation [12,13].

At present, we do not know what the best prehabilitation interventions are for patients with glioblastomas. Unlike patients waiting for major abdominal surgery, where there may be time to improve cardiovascular fitness, we do not have the same time for interventions in glioblastoma patients. Further work is needed in this area.

## 3. What Should We Resect?

Because of this pattern of glioma infiltration with individual tumor cells extending into the surrounding brain, surgery is rarely curative. The question is what do we need to remove to prolong survival and prevent the deterioration caused by tumor progression?

The contrast-enhanced tumor is the obvious target for surgery. It is easy to assess with post-operative imaging and is considered the ‘active tumor’. Numerous prospective studies show that ‘gross total resection’ of glioblastomas provides a better survival advantage than subtotal/partial resections [14,15,16]. In a retrospective survey of 416 patients, Lacroix et al. showed that a 98% resection was required to demonstrate a clear survival advantage [15]. Others suggest a stepwise improvement in survival as the extent of resection increases from a threshold of 78% resection [16]. A meta-analysis confirms that gross total resection improves overall and progression-free survival compared to subtotal resection [17].

### 3.1. Extent of Resection vs. Residual Disease

The problem with the extent of resection is that it does not account for the tumor size. A 50% resection of a 15 mL tumor leaves less tumor (7.5 mL) than a 90% resection of an 80 mL tumor (8 mL residual). A smaller volume of residual tumor is a better predictor of survival [18].

The question is whether there is a threshold of residual tumor. One study from the ALA study group demonstrated that there was only a survival advantage in patients with no residual contrast-enhancing disease [19]. There was no survival advantage to leaving less than >0 to ≤1.5 cm residual compared to >1.5 cm residual. This suggests the extent of resection of contrast-enhancing tumor needed to achieve a complete resection with no residual enhancing tumor.

### 3.2. How Do You Resect All of the Contrast-Enhancing Tumor?

The challenge is how to take the contrast-enhancing tumor on an MRI image into the operating room as our target for resection. Neuronavigation is frequently used, but early studies have failed to show any improvement in the extent of resection [20]. Neurosurgeons are poor judges of the extent of resection. In one study, neurosurgeons felt there was no residual in 71% of patients. Objective assessment of residual contrast-enhancing disease with early MRI demonstrated no residual in 18% of patients [14]. Complete resection of enhancing tumor is only performed in less than 30% of patients without adjuncts [14,21].

5-aminolevulinic acid (5-ALA) is a natural substrate of the heme biosynthesis pathway that undergoes intra-tumoral conversion into protoporphyrin-IX, a fluorophore that fluoresces in blue light. Protoporphyrin-IX accumulates in tumors, thus improving the differentiation of tumor tissue from non-tumor tissue (where there is no accumulation) [22]. A multicenter, randomized trial of 5-ALA vs. white light resection showed an improved complete resection of enhancing tumor with 5-ALA (65% vs. 29%) [23]. These results are similar to an observational study (VISIONA Study) [24], but are likely to represent early experience of 5-ALA. A recent study comparing 5-ALA to intraoperative MRI (iMRI) shows that complete resection of the enhancing tumor is seen in 78% of patients, which likely represents current practice.

Complete resection of fluorescent tumor not only encompasses the contrast-enhancing tumor component but goes beyond this [25]. Studies suggest that 5-ALA fluorescent tumor correlates with areas of increased uptake of amino acid PET in glioblastomas [26,27,28].

The issue with 5-ALA is that although there is a high positive predictive value (99%), the negative predictive value (i.e., no fluorescence means no tumor) is low at about 40%. This suggests there will be occult residual tumor.

### 3.3. Resections Beyond the Contrast-Enhanced Tumor

Even when we resect all of the fluorescent tumor (and hence all of the contrast-enhancing tumor), our data shows that 90% of patients still progress adjacent to the resection cavity. Resections that extend 1–2 cm beyond the obvious tumor reduce this local recurrence rate to 67% [29]. Patients who progressed in distant sites (i.e., where local control was achieved) did so later (21 months vs. 9 months for local recurrence), with a median survival improvement of 19 months. Similarly, improved survival is seen in patients who undergo tumor resection with lobectomy [30].

Surrounding the contrast-enhancing tumor is an area of increased FLAIR signal. Traditionally, this has been considered to be ‘edema’ or possible invasive tumor. This is no longer the case. Biopsies of this non-enhancing tumor show it has more viable tumor cells than the contrast-enhancing tumor [31].

Studies have shown that ‘supramaximal’ resections extending into this non-enhancing tumor improve outcomes. A large, retrospective study of over a thousand patients showed that resection of more than 53.21% of the FLAIR abnormality beyond the contrast-enhancing tumor demonstrated improvement in survival with no increase in neurological deficits [32]. A meta-analysis of supramaximal resection has shown a 6.4-month survival advantage compared to just resecting the enhancing tumor [33]. One difficulty is understanding what we mean by ‘supramaximal resection’ [34]. The RANO-RESECT group developed standardized definitions of the extent of resection of tumors [35]. These are summarized in Table 1. Their supramaximal contrast-enhanced resection (Class 1) provided improved overall survival (24 months vs. 19 months) and progression-free survival (11 months vs. 8 months) compared to maximal contrast-enhanced resection (Class 2). This data has led some to conclude that we should consider a ‘FLAIRectomy’ in all patients with glioblastoma [36].

There are problems undertaking such ‘FLAIRectomy’. Firstly, resecting these regions completely and avoiding damaging the normal brain is complicated. In unselected cohorts from randomized controlled trials, the rate of supramaximal resection is only 18% [35]. A recent meta-analysis of retrospective studies of supramarginal resections showed that this figure may be even lower between 4.5–50% [38]. Still, in these studies, surgeons did not state that supramaximal resection was a goal. In studies where surgeons actively pursued a supramaximal resection strategy, the number of patients eligible increased to between 39% and 47% [39,40]. This might be achievable for tumors in lobes where a lobectomy, in addition to resection of the contrast-enhanced tumor, may be possible. Numerous retrospective studies suggest an improvement in survival [39,41,42,43]. A meta-analysis of these studies suggests an improvement in progression-free survival (lobectomy 15.4 months vs. 7.3 months for gross total resection) and overall survival (25.1 months vs. 12.1 months) [30]. Lobectomy seems safe, with no increased morbidity reported [44]. All this data is based on often single-center retrospective data, but this might be a suitable area to explore further in a multi-center surgical trial.

Finally, FLAIR is not specific for tumor. A careful study of the MD Anderson Data on FLAIR resections suggests that in 18% of cases, there is a negative extent of resection of the FLAIR area. This is obviously not adding more tumor, but due to the non-specific nature of the FLAIR signal. Increased FLAIR signal can be due to tumor, edema, or even scarring from treatment effects. However, there have been attempts to separate ‘tumorous-FLAIR’ from ‘edematous-FLAIR’ [36,45].

Since FLAIR does not show us what is genuinely a tumor, we need to explore advanced imaging methods that can detect pathological changes in tumors.

### 3.4. Imaging Occult Tumor Invasion

**Figure 2:**
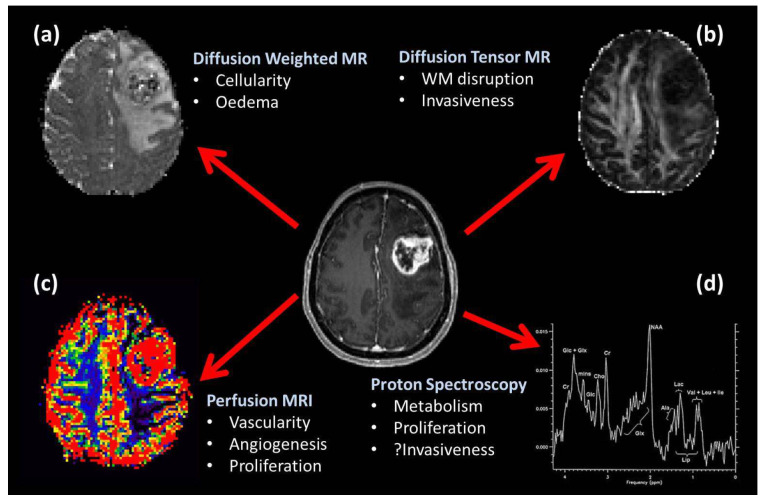
Summary of commonly available advanced MRI techniques and the pathological processes they can probe. (**a**) represents diffusion-weighted MRI, (**b**) is diffusion tensor, (**c**) is perfusion MRI and (**d**) is proton spectroscopy.

There has been much interest in using advanced imaging techniques that serve as sensitive biomarkers of pathological processes occurring within glioblastomas [46]. Figure 2 summarizes the different, commonly available imaging modalities biomarkers for tumor pathological processes.

One approach to assessing the ability of imaging biomarkers to detect occult tumors using image-guided biopsies. The methodology for these studies has been recently published [47]. Using the same methods, our group showed that diffusion tensor tissue signatures could accurately identify tumors and tumor invasion with a sensitivity of 98% and a specificity of 81% [48]. The FRONTIER study used similar methods but looked at multiple MRI and PET imaging methods alone and in combination (their findings are summarized in Table 1). The combination of ADC from diffusion MRI and O-(2-[^18^F]-fluoroethyl)-L-tyrosine PET imaging (FET-PET) provided the best sensitivity for glioblastomas [37]. Using this data, they produced maps of tumor probability intended for direct treatment.

The issue of identifying the extent of tumor invasion is that we know invasive cells permeate the whole brain [49]. Chang et al. concluded in their study about defining radiotherapy volumes that “because glioblastoma demonstrates an aggressive local recurrence pattern, it is not as important to determine where all microscopic tumor cells are residing as it is to predict where glioblastoma is most likely to recur” [50]. Several studies have looked at this with different imaging modalities.

#### 3.4.1. FET-PET Imaging

Studies with FET-PET have shown that sites of residual FET-PET increased uptake predict sites of tumor progression in 79% of patients [51]. Complete resection of the PET abnormality with no residual seen on post-operative FET-PET imaging is associated with longer survival [52].

#### 3.4.2. MR Spectroscopy

Areas with an increased Choline (Cho) to N-acetyl–aspartate (NAA) ratio are known to represent aggressive tumor. Studies have shown that regions with residual increased Cho/NAA predict where chemotherapy will progress [53]. Other studies have shown that an increased ratio of lactate/NAA can also predict the site of glioblastoma progression with a sensitivity of 88.8% and specificity of 97.6% [54].

Traditionally, MR spectroscopy has suffered with limited brain coverage and large voxels. New MR spectroscopy methods provide whole-brain coverage with voxels measuring at least 5 mm × 5 mm × 10 mm. Rivera et al. classified voxels as normal brain or areas of future progression in the FLAIR region. Using a supervised machine learning method to look at differences in the MR spectra from these regions, they identified the Choline-to-creatine (Cho/Cr) ratio as the best method. They could predict sites of tumor progression up to 8 months before progression [55]. The issue with using spectroscopy is the long acquisition times (often around 20 min) and complexity of data analysis.

#### 3.4.3. Diffusion Tensor MRI

As diffusion tensor MRI is sensitive to subtle disruption of white matter tracts, it has been suggested to be the most sensitive method of detecting occult tumor [56]. Two recent systematic reviews have confirmed that DTI is a promising biomarker for predicting sites of glioblastoma progression [57,58].

The issue with most of these studies is that they are retrospective studies developed from single units. The PRaM-GBM (NCT03294434) study is a prospective, multicenter, pragmatic study to qualify diffusion tensor metrics to identify sites of tumor progression. The favored approach appears to be using the *pq* tissue signature analysis method [59]. Using this data, we found that the extent of resection of the anisotropic component (*q*) correlates with survival [60]. In the cases where all of the *q* abnormality was resected, local control was achieved, and all patients progressed in a distant site with median survival of 31.3 months.

#### 3.4.4. Combining Imaging Modalities and Machine Learning

Machine learning combines data from different imaging modalities and training algorithms to differentiate areas of tumor progression from regions where the tumor does not progress.

Current machine learning approaches are ‘black boxes’, so we do not know how the model arrives at its conclusions or predictions. This makes it difficult to detect potential biases. There is now interest in ‘explainable’ AI, an approach that combines machine learning with modeling to build a ‘grey box’ approach.

Implementing machine learning into routine practice is difficult. A few examples of machine learning processing and analysis of medical images are available for routine clinical use. Many studies use scripts written by post-doctoral researchers that are not CE-marked. Work is needed to show how to develop machine learning tools using the correct regulations.

#### 3.4.5. Getting Advanced Imaging into the Operating Theatre

There is a problem with how to visualize the image-determined region intraoperatively. Currently, no methods exist for translating 2D imaging data into the 3D intraoperative field. Menna et al. concluded that “while the prospect of a surgical procedure guided by DTI risk maps is hopeful, it remains a distant one. The transition from 2D MRI data to the surgical field involves complex challenges and potential errors, and as of now, there have been no reports of the practical implementation of such a patient-tailored surgical technique” [57]. Image guidance methods can help, but brain shift is a major issue [61]. Methods to update this using intraoperative ultrasound, intraoperative CT, or intraoperative MRI are likely to hold the key [62].

## 4. What Not to Damage?

Unlike other parts of the body, the brain has no redundancy. We cannot resect gliomas with a normal tissue margin without damaging the brain and causing neurological deficits. Developing new motor or language deficits significantly impacts quality of life and survival [63] but can be abrogated with preoperative imaging and intraoperative monitoring [64].

### 4.1. Motor Function

Motor function has the least redundancy in the brain, but the pathways are well understood. Identifying the pre-central gyrus on MRI is straightforward but complicated when a tumor in that location distorts the brain or, more laterally, towards the Sylvian fissure. Functional magnetic resonance imaging (fMRI) provides non-invasive localization and lateralization of specific brain functions by measuring local hemodynamic changes coupled to neuronal activation. Using the differential magnetic properties of the oxygenated and deoxygenated hemoglobin, a blood oxygen-dependent (BOLD) contrast is generated. Measuring small changes in BOLD contrast with finger or toe movement vs. rest can map the primary motor cortex. More complex paradigms, where patients rehearse complex movements with and without movement, can identify structures involved in motor planning, such as the supplementary motor area [65]. Studies have shown good concordance with the gold standard of cortical stimulation, with a sensitivity of 87% and a specificity of 88% [66]. This concordance is worse with glioblastomas than low-grade gliomas (sensitivity is 65% in GBM vs. 93% in low-grade gliomas) [66]. A study of the utility of fMRI for motor mapping demonstrated that it helped assess the feasibility of tumor resection in over 50% of patients; some used it to plan surgery, and most (78%) found it helpful in planning which patients needed intraoperative neurophysiology mapping [67].

**Figure 3:**
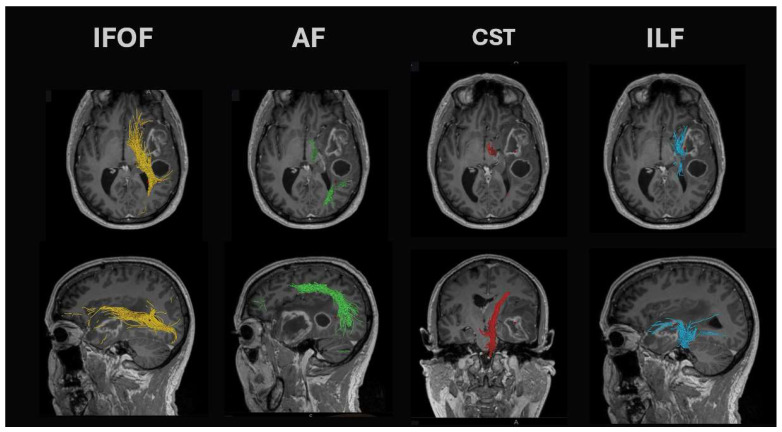
Examples of DTI tractography around a left temporal lobe glioblastoma. You can see the relationship of the tumor to the IFOF in yellow, the arcuate fasciculus (AF) in green, the corticospinal tract (CST) in red, and the ILF in blue.

Sub-cortical motor tracts can be mapped using diffusion tensor imaging (DTI). DTI measures protons’ anisotropic (directional) diffusion along the white matter tracts, resulting in an anatomical description of white matter tractography. Seed points in the pre-central gyrus or cerebral peduncle can be used to outline the corticospinal tracts (example in Figure 3). Although these are used to plan surgery, the true utility of DTI tractography preoperatively is currently unknown and is being explored in the FUTURE-GB study (ISRCTN: 38834571). What is known is that DTI tractography alone is not enough to protect the corticospinal tracts, as iMRI studies have demonstrated significant and unpredictable shifts of the corticospinal tracts during surgery [68].

Surgical techniques to map and monitor the motor pathway intraoperatively involve awake or asleep surgery with intraoperative neurophysiology. Combining both methods can be difficult as it can be challenging to differentiate voluntary movement of the patient from stimulated movement. Awake craniotomy is particularly useful for tumors involving sensorimotor regions but may only demonstrate problems when a deficit has been caused. Asleep neurophysiology allows the mapping of both cortical and subcortical areas. Using monopolar stimulation, we can judge the distance to the corticospinal tract. A rule of thumb is that every milliamp of current required to stimulate these tracts equals 1 mm distance. For glioblastomas, surgeons often use a larger threshold to stop resecting to avoid even a temporary deficit that is better tolerated in lower-grade tumors. In our experience, a threshold of 5 mA (i.e., 5 mm) is safe for glioblastomas.

Mapping of motor association regions is more complicated. The supplementary motor area (SMA) is anterior to the precentral gyrus but challenging to identify precisely. DTI tractography of the frontal aslant tract that connects the precentral cortex and pars opercularis with the supplementary motor area can be difficult to locate. The development of a supplementary motor syndrome is common following surgery in this region. Although developing this syndrome in patients with glioblastomas prolongs hospital stay and increases the need for inpatient rehabilitation, it is not associated with worse overall survival [69]. Patients develop supplementary motor syndrome in all cases where the frontal aslant tract is disrupted. Still, half of patients with SMA syndrome had an intact frontal aslant tract, suggesting preservation does not protect against SMA syndrome [69].

### 4.2. Language Function

Language is a more complex function that involves multiple processes. Although we often consider the left hemisphere dominant for language function, we know language function can be found in the right hemisphere—especially in left-handed and multi-lingual patients. The standard fMRI tasks for language include the following:

1. Word repetition vs. baseline (involving auditory perception, word comprehension, and word production) that identifies function in the left temporoparietal junction, the left inferior frontal gyrus, and motor and premotor regions.
2. Verb generation vs. baseline (involving the processes above plus semantic association and linguistic response selection), which identifies the anterior left frontal gyrus and helps determine the dominant hemisphere for language.

Language fMRI is less sensitive than motor fMRI. The sensitivity of concordance with intraoperative language mapping is limited (22–66% for individual language tasks, but improves to 59% in combinations of language tasks) [66,70], and its reproducibility can be poor: 9–26% overlap, improving to 39–44% for combined language tasks [71].

Brain mapping methods are moving away from areas of localized function determined using task-based fMRI. Rather, they are looking at brain connections using resting state fMRI. Using this methodology, several functional networks are involved in more than motor and language functions, including other cognitive functions (e.g., executive function, attention, and salience) [72]. Studies of language function have demonstrated it can identify regions that map closely with intraoperative stimulation [73]. Future work is required to see if these can replace task-based fMRI for brain mapping.

Diffusion tensor tractography of the language networks can identify several language-related white matter tracts (examples in Figure 3):

1. Arcuate fasciculus: This tract is found in the peri-insular white matter in the circular sulcus of the insula. It links the superior temporal gyrus with the dorsolateral prefrontal and premotor cortices and is found together with the lateral SLF and inferior fronto-occipital fasciculus (IFOF). Lesions of the arcuate fasciculus lead to phonemic paraphasias, repetitions, and non-fluent ‘expressive’ aphasia.
2. Superior longitudinal fasciculus (SLF): The temporoparietal branch connects the temporal lobe (posterior inferior, middle, and some superior temporal gyrus) with the parietal cortex (angular and supramarginal gyri). White matter dissection or standard DTI tractography methods cannot separate it from the arcuate fasciculus. Damage leads to fluent aphasia.
3. Inferior longitudinal fasciculus (ILF): Connects the inferior temporal gyrus and runs posteriorly to the superior and middle occipital gyri. It runs between the optic radiation (found more medial) and the more lateral SLF. Seed points for DTI tractography are best seen on coronal views at the level of the middle/inferior temporal gyri. It demonstrates marked intersubject variability in cortical terminations. Damage can lead to problems with reading (alexia).
4. Inferior fronto-occipital fasciculus (IFOF): These fibers pass from the frontal opercular cortex through the temporal stem to run on the roof of the temporal horn back to the occipital cortex. Damage leads to (visual) semantic deficits during naming tasks.

Like motor tractography, the utility of language tractography is unknown and is being investigated in the FUTURE-GB study (ISRCTN: 38834571).

Because these structures are more diffuse and can be variable, the best way to monitor language function is to perform surgery with the patient awake and map with a bipolar stimulator. What is difficult to know is the threshold we should use before we stop resecting. Again, like motor mapping, we often consider a higher threshold in glioblastoma patients as they have less time to rehabilitate.

The difficulty with awake surgery is the need for a co-operative patient who is safe for awake surgery. There are many patients for whom awake surgery is not possible due to severe anxiety or medical issues (e.g., obesity, respiratory problems, or obstructive sleep apnea). There are new attempts to map language function in asleep patients using cortico-cortical stimulation. Essentially, cortical stimulation is applied to the inferior frontal gyrus, and cortico-cortical-evoked potentials are recorded in the superior temporal gyrus [74,75] or vice versa to map the inferior frontal gyrus [76]. Mapping using this method correlates well with standard awake mapping [75], and changes in these cortico-cortical-evoked potentials provide early warning for poor language function outcomes [74,75]. More work is required to become accepted as a routine management strategy for asleep tumor resection around language areas.

### 4.3. Visual Function

The visual pathway within the brain is well understood, with structures in the temporal, parietal, and occipital lobes at risk from surgery. Although little is known about visual field loss from glioma surgery, more is known about brain injury and strokes [77,78]. Patients with hemianopias are at risk of walking into objects, tripping, and falling. They feel unsafe, rely on help in their daily activities, and have navigation problems leading to them getting lost. Loss of peripheral vision makes objects appear suddenly, causing panic in crowds and isolation. We do not know if surgically induced visual deficits persist. Spontaneous recovery is seen in 40–67% of stroke patients [79,80]—virtually always within the first month [80]. In addition, lesions can frequently lead to partial deficits and can be compensated with head and eye movements and eye tracking to minimize the effect of visual field deficits [81]. These factors mean that initial deficit and functional problems may not be long-lasting and worth experiencing to allow maximal tumor resection. The SIND study (NCT04007185) aims to understand visual field deficits’ impact on quality of life and will answer the question if we should avoid such deficits to maintain quality of life.

### 4.4. Cognitive Function

Cognitive defects affect patient functioning and independence [82] and affect patients, families, and friends. As more widespread anatomical structures are involved in these processes, they are likely at significant risk during surgery.

We have two problems assessing cognition in glioblastoma patients. The first is the lack of data on cognitive function in these patients. Although several studies report cognitive function in these patients, they are commonly part of a mixed glioma population, including low-grade gliomas and glioblastomas. A systematic review showed marked heterogeneity between studies [83], making it difficult to conclude cognitive function in glioblastomas alone.

The second issue is testing for cognitive deficits. This is usually time-consuming and can be problematic for patients who suffer from fatigue. There is also no agreement as to the best tests. Our systematic review identified 114 cognitive tests used in 432 patients: the most commonly used test was only used eight times [83]. We have used an app-based testing system that uses standardized tests on a tablet computer. This makes the testing quicker and more acceptable to patients [84] and allows for the testing of glioblastoma patients.

Using this app-based assessment tool in a cohort of glioblastoma patients tested before surgery, immediately after surgery, and before the start of radiotherapy, we found that deficits across multiple domains were common in glioblastoma patients before surgery. Following surgery, the cognitive function across all domains deteriorates before improving back to baseline before the start of radiotherapy [85]. This data shows us that cognitive deficits are common, occur before we ever see the patient, and do not seem to be made permanently worse following surgery. It also shows that if glioblastomas could be cured tomorrow, patients would still suffer from cognitive deficits.

We understand their anatomical pathways for motor, language, and visual functions to help plan surgery and avoid injury. The question is can we localize the anatomical substrates of cognitive functions? We looked at the pathways affected in patients with emotional recognition problems. We found patients had deficits of the right inferior longitudinal fasciculus, the right inferior fronto-occipital fasciculus, and anterior thalamic radiation [86]. These right-sided tracts were affected even in patients with left-sided tumors. This suggests the standard anatomical model of a region of cortex connected to a white matter tract is too simplistic for cognitive function. Instead, these complex functions are underpinned by a neural network or a connectome. We can image the functional network (using resting-state functional MRI) [87] and the structural network (using diffusion tensor MRI) [88] and understand how tumors disrupt these networks.

Disruption of the connectome likely leads to specific cognitive deficits. We know this occurs in dementia, and network disruption is seen early in the disease [89]. We do not understand how network disruption relates to specific cognitive deficits. Further research is needed.

Another unanswered question is whether patients differ in their resilience to cognitive decline. Again, we can learn from the dementia literature. It is well recognized that the extent of pathology in dementias does not correlate with cognitive decline—some patients have marked pathological changes without clinical problems [90]. This reflects differences in resilience to insults. This cognitive reserve affects inter-individual differences in task processing, allowing some individuals to cope better with injury than others. It is related to education, IQ, and lifestyle and can be estimated using questionnaires [91]. The efficiency of functional network connectivity reflects biological correlates of cognitive reserve [92].

## 5. Which Patient?

The question we must ask to allow personalized surgery is whether supramaximal surgery is beneficial for an individual patient. As one of a surgeon’s primary roles is to deliver patients to their oncologist in the best state possible to allow essential adjuvant therapy, it is worrying when a survey of the patients suitable for maximal resection found that 63% proceeded to radiotherapy with concomitant temozolomide, yet only 10% had a postoperative complication [93]. Furthermore, 27% of patients underwent maximal resection but were unsuitable for maximal oncology treatment. We need to understand better where more extensive surgery does not provide benefits and just increases the risks. As surgeons are known to be poor in determining performance status [94], we likely need to use objective performance measures to make decisions as part of a multi-disciplinary tumor board/team.

### 5.1. Role of Pre-Operative Neurological Deficits and Performance Status

Initial results from the 5-ALA study suggested an increased rate of neurological complications in the 5-ALA arm. Subsequent analysis showed that this increased rate was purely due to patients with pre-existing neurological deficits [5]. This study showed that patients with pre-existing neurological deficits (motor and language) who do not improve after steroid therapy are at a higher risk of developing worse deficits. In this cohort of patients, a less aggressive surgical strategy might be more beneficial for individual patients.

Similarly, it is well recognized that poor performance status (e.g., low Karnofsky Performance Status) is a poor prognostic factor for glioblastomas. We need to think carefully about how aggressive we should be with treating patients with poor performance status. Here a multidisciplinary—decision is essential to understand if patients would be suitable for adjuvant therapy, as without this therapy, surgery alone will not provide benefits for the patient.

### 5.2. Role of Age and Supramaximal Resection

The original study of radiotherapy with concomitant and adjuvant radiotherapy excluded patients over 70 from trial recruitment [95]. As such, elderly patients are not frequently considered for such treatment and tend to be treated with lower doses of radiotherapy. Molinaro et al. used a recursive partitioning analysis to determine the factors that impacted outcomes in glioblastoma patients [96]. They found that in patients over 65, complete resection of the enhancing tumor improved outcomes, but supramaximal resection did not provide any further benefit. In the younger age group, improved survival was not demonstrated with supramaximal surgery. This suggested a new paradigm that patients over 65 should have complete resection of the enhancing tumor. Still, for patients under 65, we should consider the non-enhancing tumor as part of our target.

### 5.3. Molecularly Defined Tumor Subtypes

We now sub-classify tumors based on molecular biomarkers. IDH mutant tumors with co-deleted 1p19q (i.e., oligodendrogliomas) are more responsive to chemotherapy. Studies have shown that leaving residual tumor does not impact survival in this group of patients. There was a significant survival advantage with more extensive resections in tumors with intact 1p19q (i.e., astrocytomas) [97].

For glioblastomas, MGMT-methylated tumors are also more sensitive to chemotherapy than unmethylated MGMT tumors [98]. Recent data suggests that leaving residual enhancing tumors in MGMT-methylated tumors does not impact survival [99]. In MGMT, unmethylated tumors, however, leaving residual tumor has a significant impact on patient survival. This data suggests that our surgery aims to differ depending on the tumor’s MGMT methylation status.

The difficulty is knowing during surgery if the patient is MGMT methylated or unmethylated. Rapid diagnosis with methylomics can now be achieved using Nanopore technology [100], which may provide the MGMT methylation status during surgery. The alternative is to use imaging and radiomics to predict the MGMT methylation status preoperatively. Currently, methods have been described that have an accuracy of 80% [101].

### 5.4. Using Prognostic Factors in Shared Decision Making

One question is how we should use this prognostic information for individual processing. The obvious approach is to discuss options with patients. Shared decision-making (SDM) is a collaborative approach in which patients and healthcare providers determine treatment decisions by incorporating clinical evidence, patients’ preferences, values, and priorities [102]. SDM can consist of four stages: First, the patient is informed that a medical decision needs to be made, and their opinion is essential. Second, the medical provider explains the risks and benefits of each treatment option. Third, a discussion occurs in which the patient and medical provider consider the patient’s preferences and support the patient. Lastly, the medical provider and patient discuss the decisional role of the patient, either make or defer the medical decision, and determine follow-up [103].

Incorporating SDM has several advantages, which include an improvement in communication, an increase in patient compliance with medical treatment, and a decrease in their decisional conflict and anticipatory anxiety. A better rapport between the medical provider and patient is established, and patients are more knowledgeable of their medical condition and empowered to make well-informed decisions [104,105]. We do not know how to include prognostic information and tools into SDM. Further work is needed to determine the potential impact of incorporating SDM aids such as decision boards, audiovisuals and information brochures, and SDM training for medical professionals.

## 6. Optimizing Post-Operative Status Before Adjuvant Oncology Treatments: Personalizing Rehabilitation

Rehabilitation has been defined as a “problem-solving educational process aimed at reducing disability and handicap (participation) experienced by someone as a result of disease or injury”. There is a common perception that brain tumor patients, especially those with limited survival, do not have rehabilitation potential. Yet, evidence suggests that brain tumor patients significantly improve function with rehabilitation [106,107,108,109,110,111,112,113,114,115,116,117,118], which leads to improvements in community independence [106] and patient survival [113]. The functional improvement is comparable to patients who underwent rehabilitation for stroke [107,114,116] and head injury [108,109], but with shorter lengths of stay [107,109]. Functional improvements are independent of tumor grade [111,113,115], although there were better initial diagnosis results than progression [111]. It appears improvements in quality of life take longer than functional improvements [110], although other studies suggest rehabilitation influences the patient’s perception of quality of life [112]. Significantly, rehabilitation does not aggravate fatigue—a common problem in brain tumor patients that impacts quality of life [117].

### 6.1. Lack of Evidence of Rehabilitation in Neuro-Oncology

The Cochrane Review 2015 identified only one interventional trial of multi-disciplinary rehabilitation for brain tumors [119]. This single-center study was not randomized; it compared a cohort of patients undergoing individualized multidisciplinary rehabilitation over 6–8 weeks over a treatment period with a control group on a waiting list [118]. They found a significant difference in measures of functional independence (the functional independence measure—FIM) at 3 months, which was maintained for 6 months. This multi-disciplinary rehabilitation was a bit of a ‘black box’, and it was difficult to know which interventions worked. They concluded that “more research in the effectiveness of ‘specific’ rehabilitation interventions is needed” [118].

### 6.2. Cognitive Rehabilitation for Brain Tumor Patients

Studies of stroke rehabilitation have identified four barriers/facilitators to deliver cognitive rehabilitation [120]:

1. Cognitive screening: A simple screening tool to identify specific cognitive deficits in brain tumor patients is needed. Computerized testing using validated cognitive measures. We have demonstrated that such tools are suitable for use and acceptable for brain tumor patients [84].
2. Cognitive rehabilitation: No one size fits all. It is essential to individualize rehabilitation to the patient and their deficits and include patients in any service. Cognitive impairment was described as a significant barrier to rehabilitation, so the interventions must overcome these problems. Some rehabilitation tools developed for specific indications may not be suitable for patients with certain patterns of cognitive deficits.
3. Psychology: A significant barrier to cognitive rehabilitation is the lack of trained neuropsychologists. Any intervention to be delivered must account for this and not rely on major neuropsychological input.
4. Joining the dots in the community: Rehabilitation services tend to be delivered in hospitals without being extended into the community. This, together with the relative rarity, means there is no expertise in managing patients with brain tumors in the community. Studies in commoner neurological disorders like stroke showed that community services are reluctant to take on stroke patients [120]. Rehabilitation cannot rely purely on community services. The survey identified in the Cochrane review concluded that their intervention trial of multi-disciplinary rehabilitation was “beyond the resources of our hospital to provide therapy for this many patients simultaneously” [118]. As a result, we need to consider how we can provide rehabilitation in patients’ homes.

Studies have shown that home-delivered rehabilitation for brain tumor patients is feasible and effective [112]. Even complex rehabilitation studies have successfully moved to online delivery during the COVID-19 pandemic [121].

## Figures and Tables

**Table 1:** Data from the FRONTIER study of the relative accuracy of the different imaging modalities looking only at IDH wildtype gliomas (The data adapted from Supplementary Figure S2 of published research of Verburg, et al. 2020 and get the permission) [37].

Imaging Type	Imaging Method	AUC (95%CI)
Conventional imaging	T1-weighted	0.58 (0.47–0.69)
	T2-weighted	0.60 (0.47–0.73)
	FLAIR imaging	0.62 (0.49–0.74)
Diffusion MRI	Apparent diffusion co-efficient (ADC)	0.66 (0.54–0.78)
	Fractional anisotropy (FA)	0.64 (0.53–0.76)
Perfusion MRI	Relative cerebral blood volume (rCBV)	0.70 (0.58–0.82)
	Relative cerebral blood flow (rCBF)	0.67 (0.56–0.78)
	Arterial spin labelled CBF (ASL-CBF)	0.53 (0.42–0.64)
MR Spectroscopy	Choline-to-NAA Index	0.64 (0.50–0.79)
PET Imaging	[^18^F]-FET	0.74 (0.62–0.86)
Combination of Imaging	ADC/FET	0.89 (0.80–0.97)

## Data Availability

No new data were created or analyzed in this study.

## Abbreviations

The following abbreviations are used in this manuscript:

5-ALA	5-aminolevulinic acid
ACSM	American College of Sports Medicine
ADC	Apparent diffusion co-efficient
AF	Arcuate fasciculus
AHP	Allied health professional
ASL-CBF	Arterial spin labeled-cerebral blood flow
BOLD	Blood oxygen level dependence
Cr	Creatine
CST	Corticospinal tract
DTI	Diffusion tensor imaging
FA	Fractional anisotropy
FET-PET	O-(2-[18F]-fluoroethyl)-L-tyrosine PET imaging
FIM	Functional Independence measure
fMRI	Functional magnetic resonance imaging
HI	Head injury
IDH	Isocitrate dehydrogenase
IFOF	Inferior fronto-occipital fasciculus
ILF	Inferior longitudinal fasciculus
iMRI	Intraoperative magnetic resonance imaging
MDT	Multidisciplinary tumor board
MGMT	O6-methylguanine-DNA-methyltransferase
MRI	Magnetic resonance imaging
NAA	N-acetyl–aspartate
PET	Positron emission tomography
rCBF	Relative cerebral blood flow
rCBV	Relative cerebral blood volume
SDM	Shared decision making
SLF	Superior longitudinal fasciculus
SMA	Supplementary motor area
